# MiR-145 mediates zebrafish hepatic outgrowth through progranulin A signaling

**DOI:** 10.1371/journal.pone.0177887

**Published:** 2017-05-22

**Authors:** Ya-Wen Li, Keng-Yu Chiang, Yen-Hsing Li, Sung-Yu Wu, Wangta Liu, Chia-Ray Lin, Jen-Leih Wu

**Affiliations:** 1 Graduate Institute of Life Sciences, National Defense Medical Center, Taipei, Taiwan; 2 Institute of Cellular and Organismic Biology, Academia Sinica, Taipei, Taiwan; 3 Department of Life science, National Taiwan University, Taipei, Taiwan; 4 Department of Chemistry, Purdue University, West Lafayette, Indiana, United States of America; 5 Department of Biotechnology, Kaohsiung Medical University, Kaohsiung, Taiwan; 6 Center for Infectious Disease and Cancer Research, Kaohsiung Medical University, Kaohsiung, Taiwan; 7 Research Center for Environmental Medicine, Kaohsiung Medical University, Kaohsiung, Taiwan; 8 Lipid Science and Aging Research Center, Kaohsiung Medical University, Kaohsiung, Taiwan; National University of Singapore, SINGAPORE

## Abstract

MicroRNAs (miRs) are mRNA-regulatory molecules that fine-tune gene expression and modulate both processes of development and tumorigenesis. Our previous studies identified progranulin A (GrnA) as a growth factor which induces zebrafish hepatic outgrowth through MET signaling. We also found that miR-145 is one of potential fine-tuning regulators of GrnA involved in embryonic hepatic outgrowth. The low level of miR-145 seen in hepatocarinogenesis has been shown to promote pathological liver growth. However, little is known about the regulatory mechanism of miR-145 in embryonic liver development. In this study, we demonstrate a significant decrease in miR-145 expression during hepatogenesis. We modulate miR-145 expression in zebrafish embryos by injection with a miR-145 mimic or a miR-145 hairpin inhibitor. Altered embryonic liver outgrowth is observed in response to miR-145 expression modulation. We also confirm a critical role of miR-145 in hepatic outgrowth by using whole-mount in situ hybridization. Loss of miR-145 expression in embryos results in hepatic cell proliferation, and vice versa. Furthermore, we demonstrate that GrnA is a target of miR-145 and GrnA-induced MET signaling is also regulated by miR-145 as determined by luciferase reporter assay and gene expression analysis, respectively. In addition, co-injection of GrnA mRNA with miR-145 mimic or MO-GrnA with miR-145 inhibitor restores the liver defects caused by dysregulation of miR-145 expression. In conclusion, our findings suggest an important role of miR-145 in regulating GrnA-dependent hepatic outgrowth in zebrafish embryonic development.

## Introduction

The liver is the largest metabolic organ that maintains numerous vital functions in the body. The liver is composed of approximately 80% hepatocytes and other cell types, including cholangiocytes, Kupffer cells, stellate cells and sinusoidal endothelial cells [1]. Liver development is divided into three stages beginning with specification in the foregut endoderm and followed by hepatic differentiation and outgrowth. At the beginning of the specification stage, hepatoblasts are derived from endodermal cells for liver bud formation. Hepatoblasts then begin to differentiate into functional hepatocytes and cholangiocytes. Finally, hepatic outgrowth occurs as hepatocytes rapidly proliferate to the appropriate size for the liver [2, 3]. Many important transcription factors and growth factors including hhex, prox1, the hnf family, Fgfs, Bmps and the Wnt pathway are involved in these processes. For example, Hhex is expressed in the hepatic bud at 22–50 hours post-fertilization (hpf) [4]. Prox1 is also expressed at 22 hpf and persists through hepatic differentiation [5]. Hepatic nuclear factors (hnf1, 3, and 4) are abundantly expressed in the liver [3]. Many liver specific genes are transcriptionally activated by different HNFs that have been extensively identified for their roles in mammalian hepatogenesis. In addition, some signaling pathways mediating hepatogenesis are conserved in mammals and zebrafish. For example, hnf1b (also called vhnf1) is required for hepatic specification in zebrafish[6]. In the Wnt pathway, mutations in wnt2bb, which is expressed at 18–52 hpf, leading to delayed hepatic specification [7]. β-catenin activation is involved in hepatic differentiation and outgrowth, and is important for maintaining liver size [8].

Progranulin (PGRN), a pleiotropic autocrine growth factor, contributes to wound healing [9], frontotemporal dementia [10, 11] and tumorigenesis [12, 13]. In hepatocarcinogenesis, tumor growth and invasion is highly correlated with elevated PGRN levels [14]. PGRN is also a therapeutic target in HCC [15]. In zebrafish, there are four PGRN genes- GrnA, GrnB, Grn1 and Grn2. Zebrafish GrnA is the orthologue of mammalian PGRN based on the syntenic conservation of chromosomal localization. The physiological role of GrnA, which regulates MET signaling in zebrafish liver developmental morphogenesis, has been shown to be specifically involved at the stage of hepatic outgrowth [16].

MicroRNAs (miRNAs) are approximately 22-nucleotide noncoding RNAs that regulates gene expression by directly targeting complementary messenger RNAs and affect mRNA degradation or translational inhibition [17]. Many studies have shown that miRNAs play fine-tuning roles in developmental processes, metabolism and diseases. To study liver development, one group explored the global function of miRNAs by generating a global miRNA knockout mouse. They observed that liver mass and hepatocyte proliferative ability are increased in hepatoblast-specific DICER1 knockout mice [18]. However, which miRNAs are required for hepatocyte maturation and proliferation in the liver remains unclear. We have been interested in identifying the regulator of GrnA-mediated MET signaling. From microRNA prediction targeting GrnA using bioinformatics software, we have identified a microRNA, miR-145. miR-145 is a tumor suppresser miRNA that is significantly downregulated in many cancers and contributes to tumor cell growth [19–22]. However, the dysregulation of embryonic hepatogenesis signaling has been shown to be highly correlated with hepatocarcinogenesis [23]. The physiological role of miR-145 in hepatogenesis remains unknown.

Zebrafish are a suitable animal model for liver development studies because the stages and mechanisms of liver development are conserved between zebrafish and mammals. In the present study, we reveal that miR-145 regulates embryonic liver size by controlling hepatocyte proliferation. In this process, miR-145 directly targets the mRNA of GrnA, and GrnA rescues the miR-145-induced liver defect. Taken together, our findings indicate that miR-145 regulates GrnA-dependent hepatic outgrowth in zebrafish development.

## Materials and methods

### Zebrafish

The wild-type (AB) zebrafish (Danio rerio) and the transgenic line *Tg (fabp10*:*EGFP)* (> 3 months) were used for egg production. The approximately 30 fish of mixed sex were kept in recirculation systems (~28°C water, pH 7.5–8) in 2-litre aquaria under a 14h:10h light-dark cycle and fed twice daily. The embryos were collected using natural mating and transferred to Petri dishes filled with E3 medium (287 mg/l NaCl, 13 mg/l KCl, 48 mg/l CaCl_2_, 81 mg/l MgCl_2_, 3 ml 0.01% (w/v) methylene blue, in dH_2_O). The embryos and larvae were kept in an incubator set at 28°C under a 14h:10h light-dark period [24]. To prevent pigmentation, the embryos were treated with 0,003% PTU at 24 hpf [25]. In all zebrafish embryo experiments, tricaine methanesulfonate (MS-222) (0.168 mg/ml) was used as a zebrafish anesthetic agent. Overdose of MS-222 (200–300 mg/l) was used for zebrafish euthanasia. The animal protocols used in this study had been evaluated and approved by the Institutional Animal Care and Use Committee of Academia Sinica (AS IACUC), Taiwan.

### miRNAs prediction on zebrafish GrnA

Computer-based programs were used to predict potential miRNAs targeting zebrafish GrnA. From miRNAs prediction of *GrnA*, miR-9, miR-206, miR-731, and miR-217 on 3’ UTR were predicted by TargetScan (http://www.targetscan.org/). The miR-145 on GrnA CDS region was predicted by MicroInspector [26] and RNAhybrid [27].

### MicroRNA mimic, inhibitor injection and rescue assay

The 3 ng/embryo of control mimic (5’-UCACAACCUCCUAGAAAGAGUAGA-3’), 1.5 and 3 ng/embryo miR-145 mimic (5’-GUCCAGUUUUCCCAGGAAUCCCU-3’), 3 ng/embryo miR-9 mimic (5’-UCUUUGGUUAUCUAGCUGUAUGA-3’), 0.03ng/embryo miR-206 mimic (5′-UGGAAUGUAAGGAAGUGUGUGG-3’), 3 ng/embryo miR-731 mimic (5’-AAUGACACGUUUUCUCCCGGAUCG-3’), 3 ng/embryo miR-217 mimic (5’-UACUGCAUCAGGAACUGAUUGG-3’) and 1.5 and 3 ng/embryo miR-145 inhibitor (Dharmacon, Thermo) were injected into one-cell stage embryos. The miR-145 inhibitor is 2′-O-methyl antisense oligonucleotides that against miR-145 were synthesized by Dharmacon RNA Technologies. Zebrafish GrnA (0.1, 0.2 and 0.4 ng/embryo) mRNA was synthesized using the mMESSAGE mMACHINE kit (Ambion, USA) and grnA morpholino (0.0625, 0.125 and 0.25 ng/embryo) (Gene Tools, USA). These were co-injected with miR-145 mimic or inhibitor for the rescue assay.

### Cell culture and transfection

ZFL cells (ATCC Number: CRL-2643) from zebrafish normal liver tissue were cultured in Leibovitz’s L-15 medium containing 50% 2 mM L-glutamine (Vitacell 30–2008), 35% Dulbecco's Modified Eagle's medium (GIBCO12100), and 15% Ham’s F12 (GIBCO 21700), which was supplemented with 5% fetal bovine serum, 0.15 g/L sodium bicarbonate, 15 mM HEPES, 0.01 mg/ml insulin, 50 ng/ml EGF and penicillin/streptomycin (Invitrogen). miR-145 mimic, miR-145 hairpin inhibitor and negative-control mimic were purchased from Dharmacon (Thermo). The day before transfection, the cells were seeded in antibiotic-free medium. Transfections of plasmids and RNA duplexes were carried out using LipofectamineTM 2000 in accordance with the manufacturer's guidelines (Invitrogen).

### Construction of reporter plasmids for luciferase assays

The predicted target sequences of GrnA were cloned downstream of the renilla luciferase gene (XhoI/NotI sites) in the psiCheck-2 plasmid (Promega) and designated the psi-Grna vector. The predicted target sequences of grnA were (5’ to 3’): forward TCGAGTCAGCCGGAGACTTGGACTGGAGCAACTGGGTCAACTGGAAGC, reverse CAGTCGGCCTCTGAACCTGACCTCGTTGACCCAGTTGACCTTCGCCGG. ZFL cells (~5×10^5^) were co-transfected with miR-145 mimic (50 nM) or control mimic (50 nM) and target reporter plasmid (1.6 μg) using LipofectamineTM 2000 (Invitrogen). The transfections and luciferase activity measurements were carried out using the Invitrogen LipofectamineTM 2000 / Promega Dual-luciferase kit according to the manufacturer’s instructions. The relative renilla activity was assessed as renilla/firefly luciferase ratios.

### Immunohistochemistry and whole-mount in situ hybridization

For immunohistochemistry, fixed and paraffin-embedded embryos were sectioned and hybridized with a PCNA antibody (PC10; Abcam, UK) and using an In situ Cell Death Detection Kit, POD (Roche, Germany). For whole-mount in situ hybridization (WISH), digoxigenin (DIG)-labeled or fluorescein-labeled antisense RNA probes for grna (NM_001001949), hhex (NM_130934), sePb (XM_001923882), prox1 (NM_131405) and fabp10 (NM_152960) were generated by in vitro transcription using T7 or SP6 RNA polymerase. miR-145 5’ and 3’ DIG-labeled LNA probes (Exiqon) were used for miRNA in situ hybridization. Confocal images were obtained using a Leica SP5 confocal microscope and were analyzed using Imaris software.

### Real-time quantitative RT-PCR

The expression levels of miR-145, GrnA and MET signaling were measured in zebrafish embryos treated with control, miR-145 mimic or miR-145 hairpin inhibitor using a High Capacity cDNA Reverse Transcription kit and Power SYBR Green PCR master Mix (Applied Biosystems). Total RNAs were extracted from each sample with TRIzol reagent (Invitrogen). The reverse transcription reaction was performed with 1 μg of total RNA. Real-time PCR was performed using a LightCycler 480 Real-time PCR system (Roche) according to the manufacturer’s instructions. The levels of U6 RNA and ef1a mRNA were used to normalize the relative miRNA and mRNA abundance.

### Western blots and antibodies

ZFL cells were transfected in six-well plates with miR-145 mimic (final concentration 100 nM), miR-145 hairpin inhibitor (final concentration 100 nM) or miR-control (final concentration 100 nM). After transfection, cells were cultured for 48 hours. Intermediate samples were collected and analyzed by western blot to assess GrnA and MET expression. The lysates were hybridized with the following primary antibodies: the polyclonal anti-GrnA antibody (1:1000), which was produced using the 4MAPS peptide EWEDHKQKKPETQRTTTRPTG (corresponding to residues 244–264 of GrnA) to immunize BALB/c mice (LTK Biolab Incorporation, Taiwan); Met antibody (1:1000, sc-10; Santa Cruz Biotechnologies, USA); and GAPDH antibody (1:1000; AnaSpec, USA).

### Imaging

All 2-dimensional images were captured using Zeiss SV-11 APO Microscope and SPOT RT3 color digital camera and analyzed by SPOT image software. The liver size was quantified by measuring the fluorescence intensity in 3-dimensional images using Leica SP5 confocal microscope and analyzed by Imaris software.

### Statistical analysis

Twenty, 30 or 100 embryos (from one clutch) per experimental group were used. Three independent replicates were performed. Data were presented as mean ± SEM (standard error of mean). Two-tailed unpaired Student’s *t*-test was used to determine the significance of differences between groups (*P ≤ 0.05; **P ≤ 0.01).

## Results

### miR-145 is one of miRNAs prediction on zebrafish GrnA that affects embryonic liver growth

**Fig 1:**
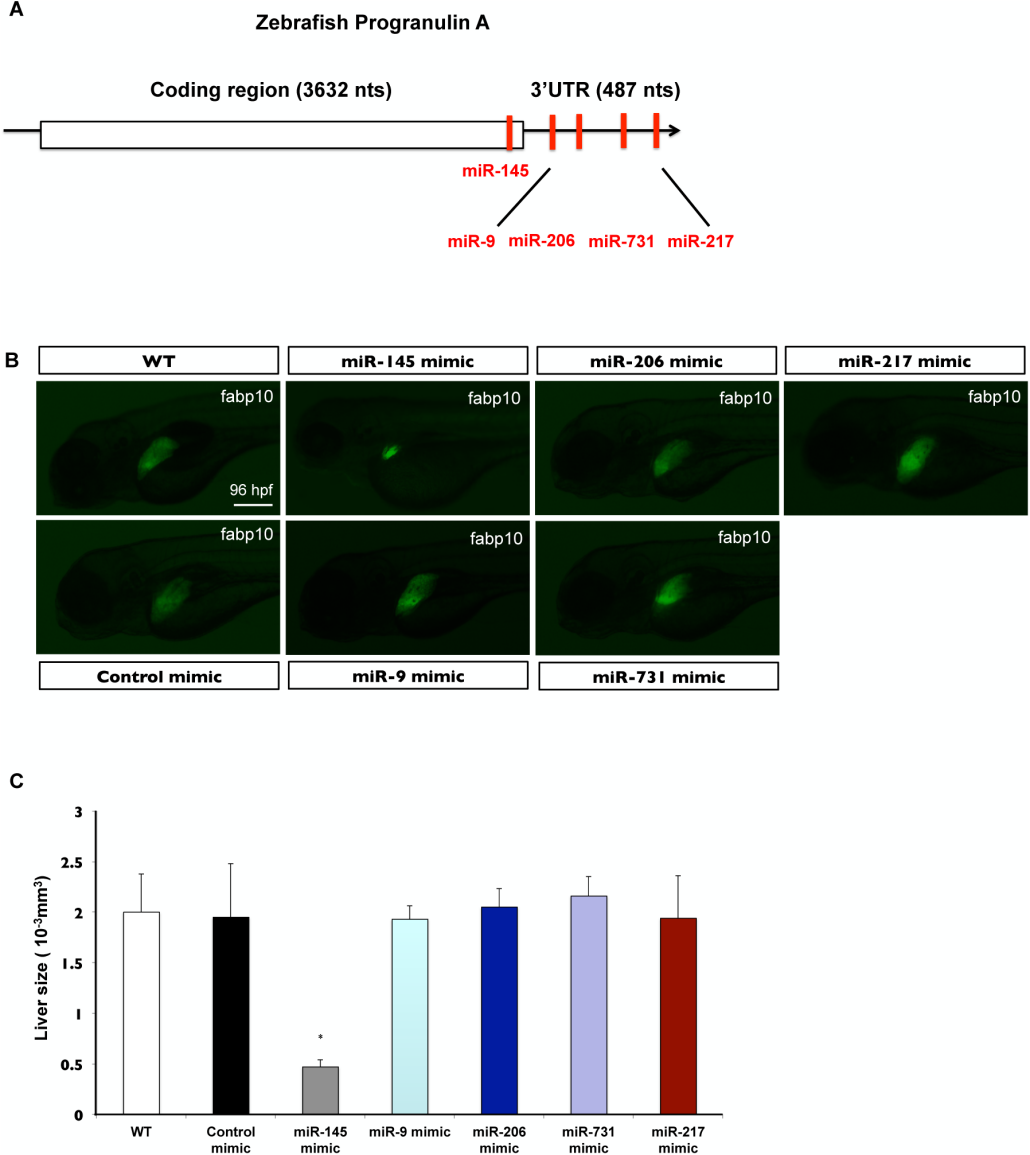
miR-145 is one of miRNAs prediction on zebrafish GrnA that affects embryonic liver growth. (A) The miRNAs prediction of GrnA. (B) Liver morphology at 4 dpf was analyzed after control mimic, miR-145 mimic, miR-9 mimic, miR-206 mimic, miR-731 mimic and miR-217 mimic injections in *Tg(fabp10*:*EGFP)* embryos. The EGFP expression of *Tg (fabp10*:*EGFP)* fish showed a smaller liver phenotype in miR-145 mimic-treated fish compared to wild type, control fish and other miRNAs mimic-treated fish. Thirty embryos per experimental group from one clutch were used and three independent replicates were performed. (C) A 3D image of the liver was analyzed using Leica SP5 confocal microscope and Imaris software. The liver size was examined by measuring the volume of EGFP expression. Ten embryos per experimental group were used and three independent replicates were performed. Scale bars, 100 μm; EGFP, enhanced green fluorescent protein; *P < 0.05, t-test.

To identify the fine-tuning regulator of GrnA-mediated MET signaling, we identified several microRNAs (miRNAs) from the miRNA predictions of GrnA using bioinformatics software. The miRNA predictions of GrnA included miR-9, miR-206, miR-731, miR-217 on 3’UTR and miR-145 on CDS region (Fig 1A). Liver morphology at 4 dpf was observed after injecting control mimic, miR-145 mimic, miR-9 mimic, miR-206 mimic, miR-731 mimic and miR-217 mimic in *Tg(fabp10*:*EGFP)* embryos. The liver size was examined by measuring the volume of EGFP expression in 3-dimension confocal images. The EGFP expression of *Tg (fabp10*:*EGFP)* fish showed no significantly changes in liver size in miR-9 mimic-, miR-206 mimic-, miR-731 mimic- and miR-217 mimic-injected fish, but the miR-145 mimic-injected fish has a smaller liver size (Fig 1B). The liver volume in miR-145 mimic-injected fish was estimated to be 0.47± 0.07×10^-3^mm^3^ and reduced to 25% liver size compared to those seen in WT (2 ± 0.38×10^-3^mm^3^)-, control mimic (1.95 ± 0.53×10^-3^mm^3^)-, miR-9 mimic (1.93 ± 0.13×10^-3^mm^3^)-, miR-206 mimic (2.05 ± 0.18×10^-3^mm^3^)-, miR-731 mimic (2.16 ± 0.19×10^-3^mm^3^)- and miR-217 mimic (1.94 ± 0.42×10^-3^mm^3^)-injected fish (Fig 1C). These findings suggest that only miR-145 can affect embryonic liver growth among the predicted miRNAs of GrnA.

### miR-145 and *GrnA* expression patterns are inversely correlated during liver development

**Fig 2:**
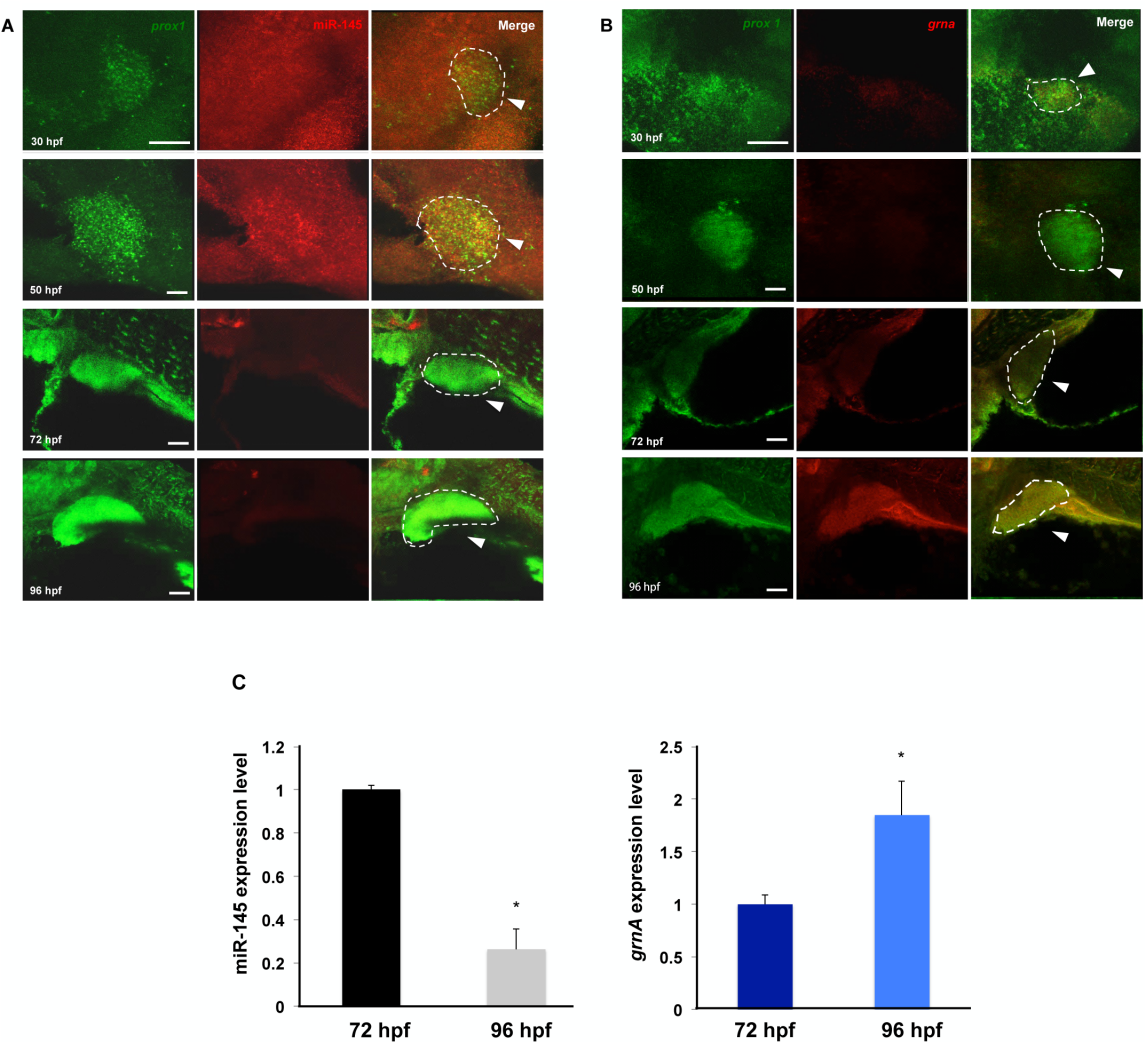
miR-145 and GrnA expression patterns are inversely correlated during liver development. (A,B) The expression patterns of miR-145 (A), *grnA* (B) and the hepatoblast-specific marker gene *prox1* at 30, 50, 72 and 96 hpf were examined using FISH in wild-type zebrafish embryos. Scale bars, 25 μm (at 30 and 50 hpf), 50 μm (at 72 and 96 hpf). The dotted circles indicate *prox1* positive cells that represent liver. Ten embryos per experimental group were used and three independent replicates were performed. (C) The fetal liver at 72 and 96 hpf were isolated to quantify liver specific expression patterns of miR-145 and *GrnA*. Fifteen fetal livers per experiment were used and three independent replicates were performed. The relative expression is normalized with internal control, U6 and *ef1a* expression.

Fluorescence in situ hybridization (FISH) was used to determine the miR-145 and *GrnA* expression patterns specifically in liver development. Fluorescence-labeled RNA probes of the hepatoblast-specific marker prospero-related homeobox 1 (*prox1*) and DIG-labeled miR-145 or GrnA RNA probes were detected at 30, 50, 72 and 96 hpf in wild-type (AB) zebrafish embryos. miR-145 was expressed in hepatoblasts at 30 and 50 hpf but decreased at 72 and 96 hpf (Fig 2A). In contrast, GrnA expression increased from 30 to 96 hpf (Fig 2B). To analyze the liver specific expression pattern of miR-145 and *GrnA*, we isolated the fetal liver at 72 and 96 hpf to perform qPCR. The qPCR result reveals that the expression of miR-145 in embryonic liver at 96 hpf decreased as compared to that at 72 hpf. Inversely, the expression of GrnA in embryonic liver at 96 hpf increased as compared to that at 72 hpf (Fig 2C). These results indicate that miR-145 and *GrnA* expression are inversely correlated during liver development.

### miR-145 expression level negatively regulates embryonic liver size

**Fig 3:**
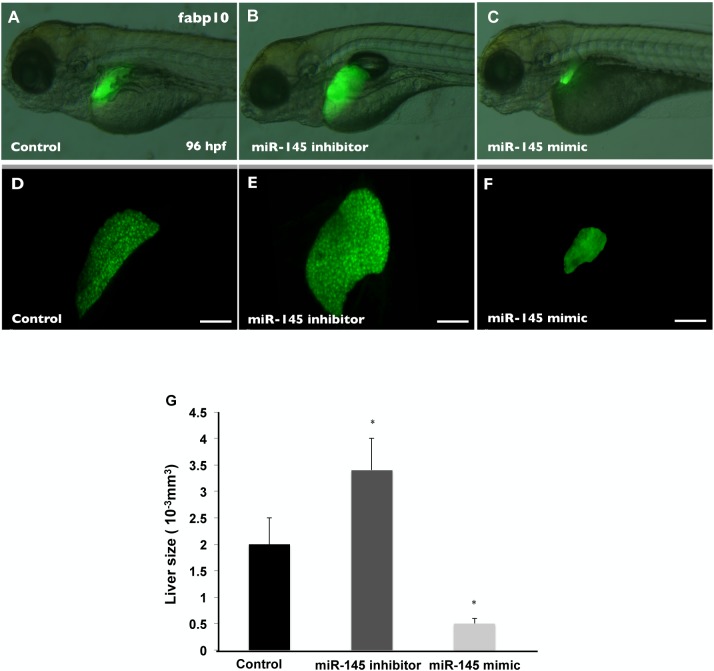
miR-145 is critical for liver morphogenesis. Liver morphology at 4 dpf after control mimic (A), miR-145 inhibitor (B) and miR-145 mimic (C) injections in Tg(fabp10:EGFP) embryos. One hundred embryos per experimental group from one clutch were used and three independent replicates were performed. A 3D image of the liver was analyzed using Leica SP5 confocal microscope and Imaris software (D-F). The liver size was examined by measuring the volume of EGFP expression (G). Ten embryos per experimental group were used and three independent replicates were performed. The EGFP expression of *Tg (fabp10*:*EGFP)* fish showed an enlarged liver in miR-145 inhibitor-injected fish and an underdeveloped liver in miR-145 mimic-injected fish fish as compared to that in control fish. Scale bars, 100 μm; EGFP, enhanced green fluorescent protein; *P < 0.05, t-test.

To study the regulatory mechanism of miR-145 in embryonic liver development, we blocked the mature miR-145 function using a miR-145 hairpin inhibitor and enhanced miR-145 expression using a miR-145 RNA mimic. To control for the specificity of the miR-145 mimic and inhibitor, a negative control mimic was used, the sequence of which was based on *C*. *elegans* microRNAs that have minimal sequence identity to zebrafish. A total of 3 ng of control, miR-145 inhibitor or miR-145 mimic was injected into wild-type zebrafish embryos. In situ hybridization using an LNA antisense probe against mature miR-145 indicated that the miR-145 inhibitor reduced the level of miR-145 (S1C and S1D Fig) and miR-145 mimic treatment increased miR-145 expression (S1E and S1F Fig) compared to that seen in control mimic-injected fish at 72 hpf (S1A and S1B Fig). In addition, quantitative real-time PCR (qPCR) of miR-145 showed that apparent miR-145 was significantly decreased after miR-145 inhibitor injection and that apparent miR-145 expression was increased approximately thirty fold after miR-145 mimic injection (S1G Fig). To test the specificity, we determined the expression level of *Gata6*, which is a known target of miR-145 [28], in miR-145 mimic- and inhibitor-injected fish. The expression level of *Gata6* showed a 2.4-fold increase in miR-145 inhibitor-injected fish and a 0.6-fold decrease in miR-145 mimic-injected fish (S1H Fig). These results suggest that the mimic and inhibitor treatments specificity and efficiently modulate miR-145 expression. To study the influence of miR-145 in liver development, the 1.5 ng and 3 ng of control, miR-145 mimic or miR-145 inhibitor was injected into *Tg(fabp10*:*EGFP)* zebrafish. The *Tg(fabp10*:*EGFP)* is a transgenic fish that has liver-specific expression of enhanced green fluorescent protein (EGFP) at 36 hpf [29]. After miR-145 inhibitor and mimic injection, the dose-dependent alteration of the size of liver expressing EGFP at 4 dpf was observed. The liver morphology in 3 ng miR-145 mimic and inhibitor treatment was significantly affected (S2 Fig). After 3ng miR-145 inhibitor injection, miR-145 inhibition significantly increased the size of liver expressing EGFP at 4 dpf (Fig 3B). In contrast, embryos with overexpression of miR-145 exhibited smaller EGFP-expressing liver compared to that seen in control-injected liver (Fig 3A and 3C). The liver size was determined by measuring the volume of EGFP-expressing livers using three-dimensional confocal images (Fig 3D–3F). At 4 dpf, the liver size of control mimic-injected *Tg(fabp10*:*EGFP)* embryos was 2.0 ± 0.5×10^-3^mm^3^. The liver size of miR-145 inhibitor-injected *Tg(fabp10*:*EGFP)* embryos increased by approximately 170% (3.4 ± 0.6×10^-3^mm^3^); however, the liver size of miR-145 mimic-injected *Tg(fabp10*:*EGFP)* embryos reduced to 25% of the liver size of the control group (0.5± 0.1×10^-3^mm^3^) (Fig 3G). These results indicate that miR-145 expression level negatively affects liver size at 4 dpf in zebrafish.

### miR-145 is required for hepatic outgrowth during liver development

**Fig 4:**
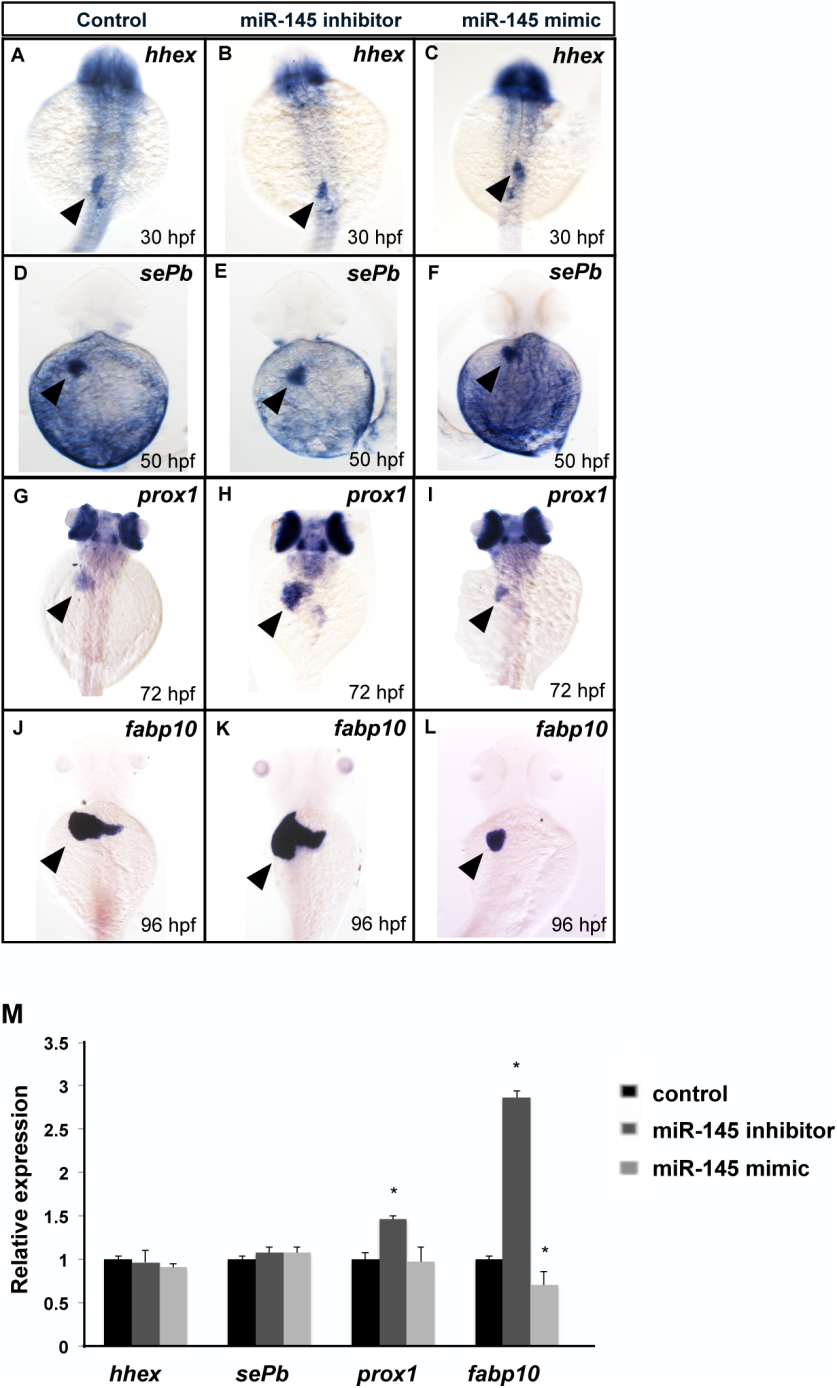
miR-145 is required for hepatic outgrowth. A WISH assay was used for determination of the liver developmental markers *hhex* at 30 hpf (A-C, dorsal view), *sePb* at 50 hpf (D-F, dorsal views), *prox1* at 72 hpf (G-I, dorsal views) and *fabp10* at 96 hpf (J-L, dorsal views) in control, miR-145 inhibitor-injected and miR-145 mimic-injected embryos. The arrowhead indicates liver. The expression levels of *hhex*, *sePb*, *prox1* and *fabp10* were examined using qPCR (M). The *ef1a* expression level was served as an internal control (*P < 0.05, t-test). Twenty whole embryos per experimental group were used and three independent replicates were performed.

Whole-mount in situ hybridization (WISH) was used to clarify the stages of liver development affected by miR-145. We detected the gene expression of the liver specification marker hematopoietically expressed homeobox (*hhex*). The miR-145 inhibitor- and miR-145 mimic-injected embryos exhibited a normal expression pattern of *hhex* at 30 hpf (Fig 4A–4C). The differentiation marker selenoprotein Pb (*sePb*) was detected at 50 hpf and also exhibited a normal expression pattern in miR-145 inhibitor- and miR-145 mimic-injected embryos as compared to those in controls (Fig 4D–4F). At the later hepatic outgrowth stage, the prospero-related homeobox 1 (*prox1*) and fatty acid-binding protein 10 (*fabp10*) were expressed in mature hepatocytes after 72 hpf. miR-145 inhibitor-injected embryos had increased *prox1* expression and miR-145 mimic-injected embryos had reduced *prox1* expression at 72 hpf (Fig 4G–4I). Furthermore, *fabp10* expression also increased in miR-145 inhibitor-injected embryos at 96 hpf, while, inversely, *fabp10* expression decreased in miR-145 mimic-injected embryos at 96 hpf (Fig 4J–4L). The quantification of mRNA expression levels using qPCR revealed that *hhex* expression at 30 hpf and *sePb* expression at 50 hpf did not exhibit significant changes under miR-145 mimic and inhibitor administration. The *proxl* expression at 72 hpf and *fabp10* expression at 96 hpf increased by 1.5-fold and 2.9-fold, respectively, in miR-145 inhibitor-injected embryos. Inversely, *fabp10* expression at 96 hpf showed a 0.5-fold decrease in miR-145 mimic-injected embryos (Fig 4M). In conclusion, these whole-mount and qPCR data indicate that miR-145 expression has major effects on hepatic outgrowth stage during liver development.

### miR-145 influences hepatocyte proliferation in zebrafish embryos

**Fig 5:**
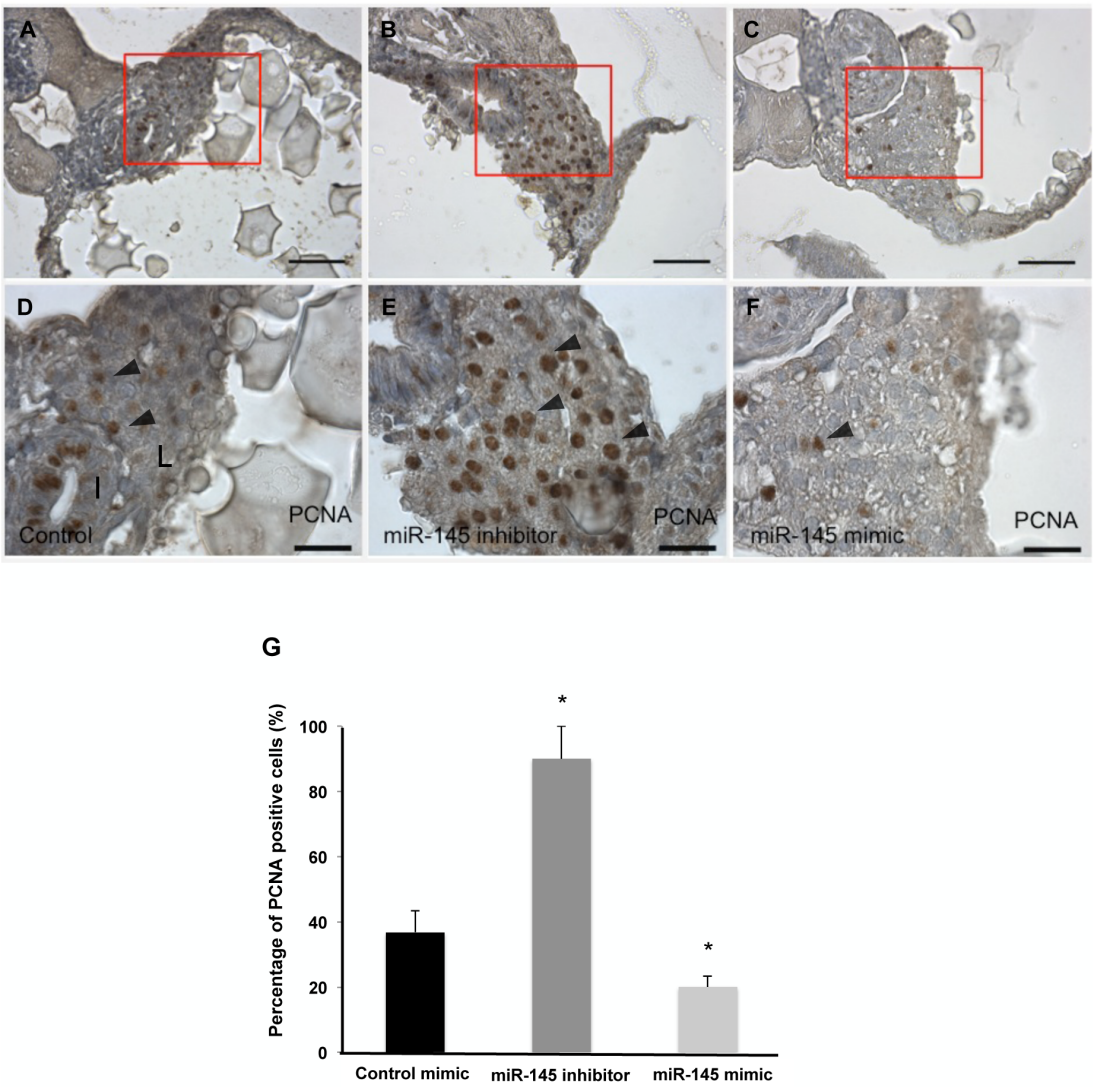
miR-145 modulates hepatocyte proliferation in zebrafish embryos. The liver sections of control mimic-, miR-145 inhibitor- and miR-145 mimic-injected fish were examined using PCNA staining at 4 dpf. The liver sections of three embryos per experimental group were used and three independent replicates were performed. miR-145 inhibitor-injected fish had more PCNA-positive hepatocytes (B, E) as compared to those in control (A,D). In contrast, a low signal was detected in response to miR-145 mimic treatment (C,F). The arrowhead indicates PCNA-positive hepatocytes. The percentage of PCNA-positive hepatocytes was counted (G). The blue staining is hematoxylin stain; I, intestine; L, liver; Scale bars, 20 μm; *P < 0.05, t-test.

To investigate whether miR-145 is involved in embryonic hepatic outgrowth through controlling cell proliferation, we performed immunohistochemistry with an antibody against proliferating cell nuclear antigen (PCNA) to observe cell proliferation in liver sections at 4 dpf in control mimic-, miR-145 mimic- and miR-145 inhibitor-injected fish. The percentage of PCNA-positive hepatocytes was increased by 2.4-fold in response to miR-145 inhibition in embryos at 4 dpf: 36.7 ± 6.7% in the control versus 90 ± 10% in the miR-145 inhibitor-injected fish (Fig 5E and 5G). In contrast, the PCNA-positive hepatocytes of embryos with miR-145 overexpression showed a 1.8-fold reduction compared to those in controls at 4 dpf: 36.7 ± 6.7% in the control versus 20 ± 3.3% cells in miR-145 mimic-injected fish (Fig 5F and 5G). These data suggest that miR-145 regulates cell proliferation and embryonic hepatic outgrowth. Consequently, miR-145 expression attenuates cell proliferation in embryonic liver.

### miR-145 directly targets and modulates GrnA expression

**Fig 6:**
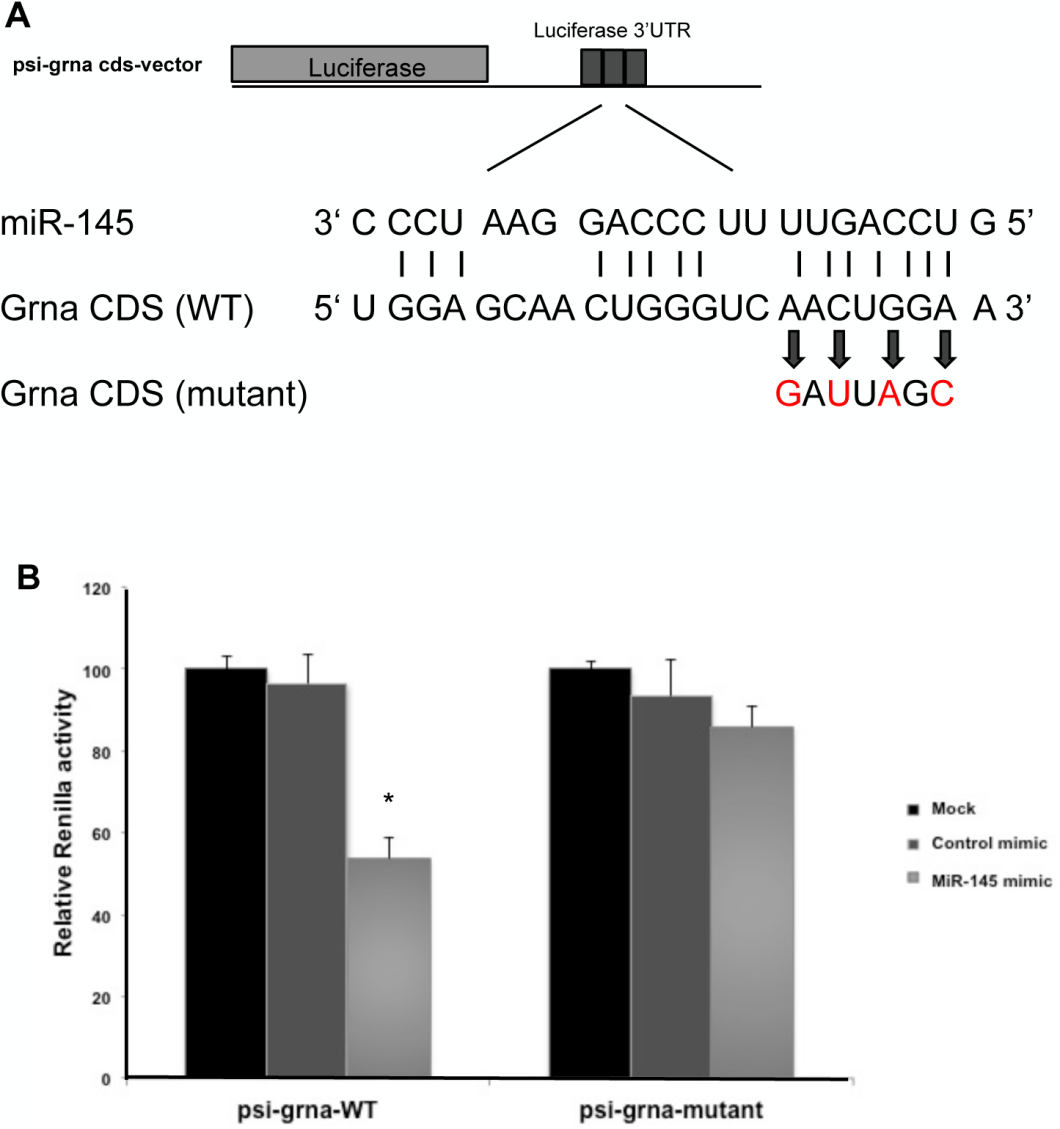
miR-145 directly targets grnA as determined by luciferase assay. The predicted miR-145 target site on GrnA CDS is illustrated. WT and mutant forms of *GrnA* CDS were constructed in *psi-check2* reporter vector (A). miR-145 mimic and *psi*-grna-WT/*psi*-grna-mutant vector were co-transfected into ZFL cells for the luciferase assay. The luciferase activity of *psi*-grna-WT was suppressed approximately 45% in response to miR-145 mimic treatment. In contrast, the luciferase activity of *psi*-grna-mutant was unchanged in response to miR-145 mimic treatment (*P < 0.05, t-test) (B). Three independent replicates were performed.

**Fig 7:**
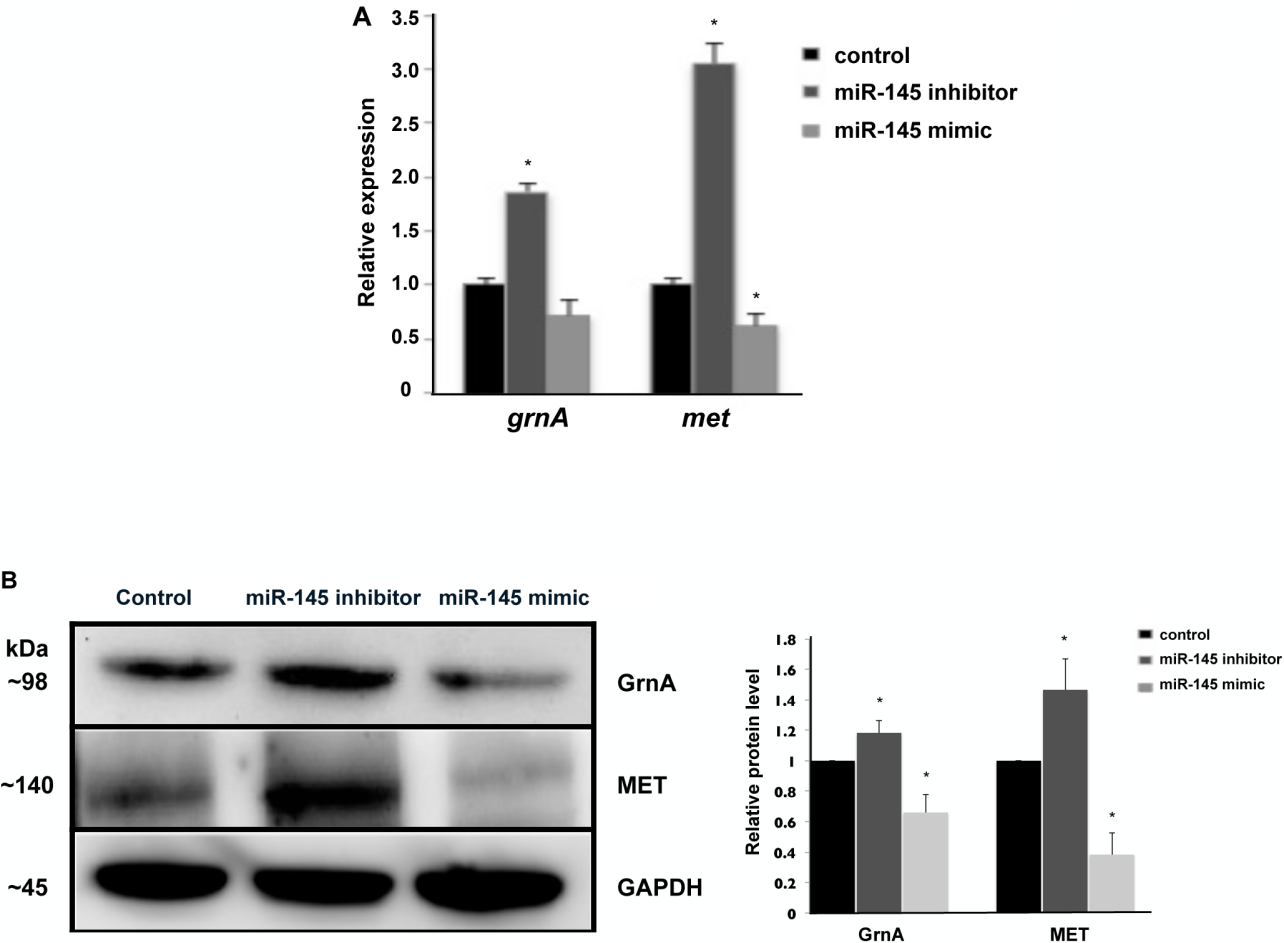
miR-145 regulates GrnA and MET gene expression. The expression levels of the *grnA* and *met* genes were examined using qPCR after 24 hours of control, miR-145 mimic and miR-145 inhibitor treatment in ZFL cells (A). The protein levels of GrnA, MET and GAPDH were examined by Western blotting in ZFL cells at 48 hours after control, miR-145 mimic and miR-145 inhibitor treatments. The relative GrnA, MET and GAPDH protein levels were quantified as shown in the low panel (*P < 0.05, t-test) (B). Three independent replicates were performed.

To determine whether GrnA is a direct target of miR-145 in mediating hepatic outgrowth, we conducted bioinformatics software prediction using MicroInspector[26] and RNAhybrid [27] to identify the putative miR-145 recognition site located in exon 20 of the GrnA. First we made a construct containing the putative miR-145 recognition site behind a luciferase reporter (*psi-grna vector*, Fig 6A). A mutant control was generated with the luciferase reporter vector followed by four nucleotides mutant at the putative miR-145 recognition site region. We co-transfected control mimic/miR-145 mimic and *psi-grna / psi-grna* mutant vector into the zebrafish liver cell line (ZFL cell line) for the luciferase assay. Luciferase activity was suppressed approximately 45% when miR-145 was co-expressed with the *psi-grna* vector, but there was no significant effect on the mutant control (Fig 6B). These data suggest that miR-145 binds the putative miR-145 recognition site of GrnA in transfected ZFL cells. Furthermore, we determine whether endogenous GrnA expression is affected by miR-145. Control mimic, miR-145 inhibitor and miR-145 mimic were transfected into ZFL cells to assess the activation of GrnA and MET signaling using qPCR and Western blotting. At 24 hours after control mimic, miR-145 inhibitor and miR-145 mimic (100 nM) treatments in ZFL cells, the mRNA expression levels of GrnA and MET increased in ZFL cells treated with miR-145 inhibitor as compared to those in control-treated cells. In contrast, GrnA and MET were downregulated in miR-145-overexpressing ZFL cells (Fig 7A). Therefore, after 48 hpf, the protein expression levels of GrnA and MET were further examined using Western blotting. Following miR-145 inhibition, the results showed that GrnA and MET protein expression levels increased by 1.2-fold and 1.5-fold, respectively, as compared to those in control mimic treatment in ZFL cells. In contrast, GrnA and MET protein expression levels reduced by 0.7-fold and 0.4-fold, respectively, in miR-145-overexpressing ZFL cells as compared with those in control mimic-treated cells (Fig 7B). These data suggest that miR-145 modulates GrnA expression through binding to GrnA mRNA in zebrafish liver.

### GrnA can rescue the defect in liver outgrowth in miR-145 morphants

**Fig 8:**
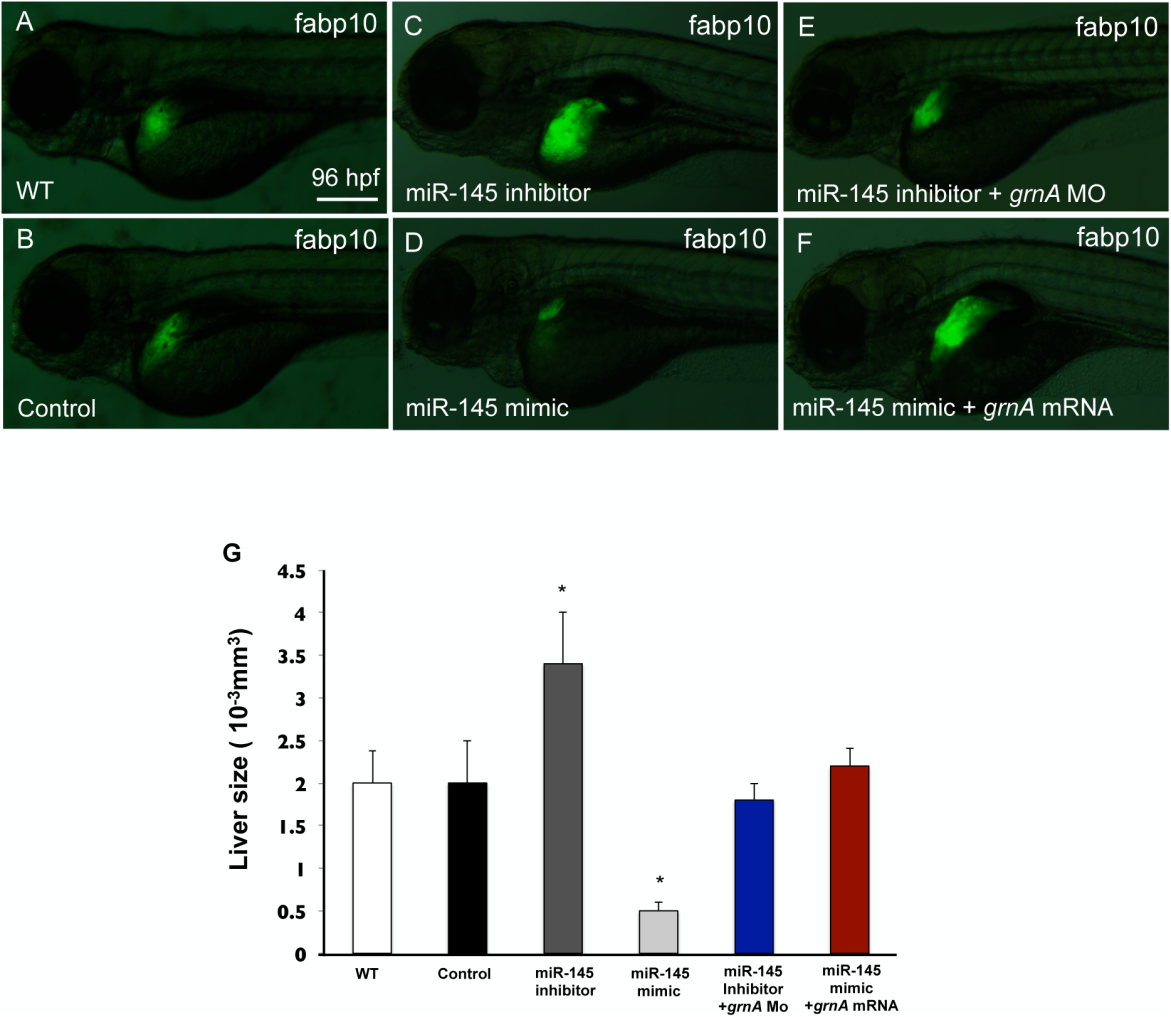
GrnA rescues the hepatic outgrowth defect caused by miR-145 manipulation. Liver morphology was determined by EGFP expression at 4 dpf in *Tg (fabp10*:*EGFP)* embryos (A) or in embryos injected with control mimic (B), miR-145 inhibitor (C), miR-145 mimic (D), miR-145 inhibitor with *grnA* MO (0.25 ng/embryo) (E) and miR-145 mimic with *grnA* mRNA (0.4 ng/embryo) (F). Thirty embryos per experimental group were used and three independent replicates were performed. A 3D image of the liver was observed using Leica SP5 confocal microscope and Imaris software. The liver size was examined by measuring the volume of EGFP expression (G). Ten embryos per experimental group were used and three independent replicates were performed. Scale bars, 100 μm; EGFP, enhanced green fluorescent protein; *P < 0.05, t-test.

To determine whether miR-145 affects embryonic hepatic outgrowth through targeting GrnA, we performed *GrnA* mRNA and morpholino rescue experiments to verify whether GrnA signaling can rescue the miR-145-induced liver defect. miR-145 knockdown resulted in a significant increase in liver size at 96 hpf in *Tg(fabp10*: *EGFP)* fish (Fig 8C). Co-injection of the miR-145 inhibitor with 0.25 ng/embryo GrnA morpholino in *Tg(fabp10*: *EGFP)* embryos restored normal liver size (Fig 8E). Smaller liver sizes were observed at 96 hpf in miR-145 overexpressing-*Tg(fabp10*: *EGFP)* fish (Fig 8D). We co-injected miR-145 mimic and 0.4 ng/embryo GrnA mRNA into *Tg(fabp10*: *EGFP)* embryos and observed that the liver size was restored to normal (Fig 8F) compared to that in the control at 96 hpf (Fig 8B). The quantification of liver volume showed enlarged liver (3.4± 0.6×10^-3^mm^3^) in miR-145 inhibitor-injected fish and smaller liver (0.5± 0.1×10^-3^mm^3^) in miR-145 mimic-injected fish as compared with normal livers in WT and control mimic-injected fish (2.0 ± 0.4×10^-3^mm^3^ and 2.0 ± 0.5×10^-3^mm^3^, respectively). Co-injection of miR-145 inhibitor with *GrnA* morpholino restored normal liver size (1.8 ± 0.2×10^-3^mm^3^); similarly, co-injection of miR-145 mimic and GrnA mRNA also restored normal liver size (2.2 ± 0.2×10^-3^mm^3^) (Fig 8G). In addition, the *GrnA* mRNA and morpholino rescued the liver size in a dose-dependent manner (S4 Fig). These results suggest that miR-145 modulates embryonic liver outgrowth through regulating GrnA signaling.

## Discussion

The regulatory mechanisms of liver development are complex, and involve in many growth factors and transcription factors across three stages-hepatic specification, differentiation and outgrowth. The miRNAs are known to be important genetic regulators in development. A liver specific miRNA, miR-122a, has been reported to control hepatocyte differentiation [30]. Besides miR-122a, little is known about the function of other miRNAs involved in liver development. Researchers have generated liver-specific DICER1 knockout mice to prove that the loss of miRNAs increases liver cell proliferation. However, the fact that which miRNAs participate in embryonic liver cell proliferation remains unclear. In a previous study, PGRN is a pleiotropic autocrine growth factor that was found to be essential for hepatic outgrowth through transcriptional regulation of MET during liver development [16]. Therefore, we are interested in exploring the fine-tuning regulator on PGRN signaling involved in embryonic liver growth. We identified several miRNAs from the miRNA predictions of zebrafish GrnA. Because the 3’UTR of GrnA is 487 nucleotides in size, there are only putative four miRNAs, including miR-9, miR-206, miR-731 and miR-217 predicted by TargetScan. In this study, we first showed the dysregulation of four miRNAs couldn’t influence liver development. The miR-9 is abundantly expressed in developing neurogenic cell that regulates neuron differentiation [31]. The miR-206 is a muscle specific miRNA participating in myogenesis [32]. The miR-217 is expressed in the pancreas [33]. The miR-731 is induced by hypoxic stress [34]. Hence, the four miRNAs may not regulate liver development. We demonstrate that miR-145, which targets the CDS region of GrnA, can affect embryonic liver growth. The expression pattern of miR-145, especially in the embryonic liver, has not been confirmed. We also demonstrate an inverse correlation between miR-145 and GrnA expression during hepatogenesis. miR-145 expression in the liver primordium decreases from 30 to 96 hpf and GrnA expression increases from 30 to 96 hpf. The expression pattern of GrnA is similar to that previously reported [35]. miR-145 is expressed in liver of zebrafish embryos at 30 hpf. miR-145 is ubiquitously expressed at 19-somite zebrafish embryos as demonstrated by Zeng et al. [28]. Furthermore, we show that miR-145 is almost not expressed in zebrafish embryos at 96 hpf. Wienholds’s and Zeng’s groups have proved that miR-145 is expressed in gut but not in liver of zebrafish embryos at 96 hpf [28, 33]. Moreover, GrnA is widely expressed in many other tissues. Its expression increases from 24 to 96 hpf [35].

Numerous reports have indicated that miR-145 is a suppressor of tumor cell proliferation in various types of cancers [36–39], including liver cancer [40]. The mechanisms of tumorigenesis have been shown to be associated with the dysregulation of embryonic signaling pathways. Although miR-145 has been shown to be involved in liver cancer progression, the functional roles of miR-145 in embryonic liver development are still unknown. This study is the first to show that the manipulation of miR-145 expression affects embryonic liver size. Expression of certain specific markers at different liver developmental stages is affected by miR-145. However, the expression of *hhex*, a marker for liver specification, is affected neither by the loss of miR-145 nor by miR-145 overexpression. At liver budding stages, *sePb* expression is also not significantly altered. In contrast, at hepatic outgrowth stages, loss of miR-145 increases *prox1* and *fabp10* expression, while overexpression of miR-145 decreases *prox1* and *fabp10* expression. We show that manipulation of miR-145 has minor effects on the expression of specification- and differentiation-related genes. However, we cannot exclude the possibilities of miR-145 involvement in hepatic specification and/or differentiation. We hypothesize that hepatocyte outgrowth is the major target of miR-145 during liver development and that the process of hepatic proliferation is activated in the outgrowth stages. We reason that loss or attenuation of miR-145 expression promotes hepatic proliferation and liver outgrowth, and inversely, miR-145 overexpression suppresses hepatic proliferation and liver growth. Interestingly, miR-145 expression is downregulated in liver cancer, and restoration of miR-145 expression in liver tumor cells results in inhibition of proliferation in these cells [21, 22]. Our results suggest that the relevant mechanisms of miR-145-mediated growth regulation may exist in both embryonic hepatic growth and liver tumor growth.

In this study, we decide to determine whether the direct target gene of the miRNA is useful for studying the biological function of miR-145 during liver development. The seed sequence between the 5’ end of the microRNA and the mRNA is a critical determinant of the potential binding site. Usually, a perfect or near perfect match of the seed sequence between miRNA and mRNA targets will lead to mRNA degradation [41]. We have found that the coding region of GrnA has a near perfect complementary binding site for the miR-145 seed region. Furthermore, in humans, PGRN is also a predicted target of miR-145. It is possible that the miR-145 and PGRN regulatory mechanism is conserved. Importantly, the results of the luciferase activity assay suggest that miR-145 directly targets *GrnA*. In a previous study, GrnA is shown to be essential for hepatic outgrowth through transcriptional regulation of MET during liver development [16]. Our findings reveal that the mRNA and protein expression levels of GrnA are inversely regulated in response to the loss and overexpression of miR-145. At the same time, MET gene is also inversely regulated by miR-145. In the rescue experiments, we show that GrnA expression rescues the hepatic outgrowth defect caused by miR-145 manipulation. Consequently, we show that miR-145 may serve as a novel regulator of GrnA to modulate hepatic outgrowth. Our results indicate an important role of miR-145 in modulating hepatic outgrowth.

In addition, we find an inverse correlation between miR-145 downregulation and GrnA overexpression in zebrafish liver outgrowth. Similar gene expression patterns of miR-145 and PGRN also occur in pathological liver growth. miR-145 downregulation and PGRN overexpression are required for liver tumor cell proliferation. PGRN is a growth factor and has been reported to be associated with the tumor growth of many cancers, such as breast [42], ovary [43], liver [14], kidney [44] and bile duct carcinomas [45]. PGRN overexpression promotes tumor cell growth through MEK/Erk and PI3K/Akt signaling activation [46]. In addition, our previous study shows that GrnA regulates Erk signaling through transcriptional activation of MET in zebrafish liver outgrowth [16]. miR-145 is downregulated and serves as a tumor suppressor microRNA in many types of cancers, such as breast [47], ovary [48], liver [19] and colon cancers [49]. In tumor growth, restoration of miR-145 inhibits the PI3K/Akt pathway and suppresses cell growth [50]. miR-145 also reduces MEK/Erk signaling in tumor cells [51]. Taken together, these results suggest that miR-145 and PGRN regulate the same downstream signaling pathways-the MEK/Erk and PI3K/Akt pathways. In this study, we show that miR-145 targets GrnA in attenuating zebrafish liver outgrowth. For further study, we propose that miR-145 targeting of PGRN may contribute to tumor cell growth in cancer progression.

Taken together, we identify a new role of miR-145 that is required for hepatic outgrowth through regulating GrnA expression. Moreover, we provide a zebrafish model that could be used to study the regulatory mechanisms of miRNAs and their target mRNAs during liver development. The regulatory mechanism of miR-145 and GrnA is important not only in physiological liver growth but also in pathological liver growth. This mechanism may be a new therapeutic target for liver cancer.

## Supporting information

S1 Fig miR-145 mimic and miR-145 hairpin inhibitor treatment specificity and efficiently regulate miR-145 expression in vivo. The miR-145 expression pattern at 72 hpf was examined using WISH and qPCR in control-, miR-145 inhibitor- and miR-145 mimic-injected fish. At 72 hpf, WISH indicates normal miR-145 expression in control-injected fish (A and B), reduced miR-145 expression in miR-145 inhibitor-injected fish (C and D) and overexpression of miR-145 in miR-145 mimic-injected fish (E and F). (A, C and E are dorsal views; B, D and F are lateral views). The qPCR analysis reveals that the miR-145 mimic and inhibitor modulate miR-145 expression (G). In addition, the qPCR analysis reveals that *gata6* expression is regulated by altered expression of miR-145 (H). The *ef1a* and U6 expression was measured as a loading control. (**, *P < 0*.*01*, *t-test*; the arrow indicated liver, thirty embryos per experimental group were used and three independent replicates were performed).
(TIFF)

S2 Fig The dose-dependent effects of miR-145 mimic and inhibitor on hepatic outgrowth. Liver morphology at 4 dpf after 1.5 ng and 3 ng control mimic, miR-145 inhibitor, miR-145 mimic injection in *Tg(fabp10*:*EGFP)* embryos. (Scale bars, 200 μm; EGFP, enhanced green fluorescent protein; thirty embryos per experimental group were used and three independent replicates were performed).
(TIFF)

S3 Fig The developmental growth is normal under altered expression of miR-145. Two mesodermal genes, heart and neural crest derivatives expressed transcript 2 (*hand2*) at 30 hpf and myogenic differentiation 1(*myod*) at 54 hpf was examined using WISH. The result reveals mesoderm development was not significantly affected by manipulation of miR-145 expression (A). The body length were measured at 96 hpf after control mimic, miR-145 inhibitor, miR-145 mimic injection in *Tg(fabp10*:*EGFP)* embryos (B). (Scale bars, 0.50 mm; thirty embryos per experimental group were used and three independent replicates were performed).
(TIFF)

S4 Fig The three doses of GrnA rescue the hepatic outgrowth defect caused by miR-145 manipulation. Liver morphology was determined by EGFP expression in *Tg(fabp10*:*EGFP)* embryos at 4 dpf (A) or in embryos injected with control mimic (B); miR-145 inhibitor (C); miR-145 mimic (D); miR-145 inhibitor with three doses of *grnA* MO 0.0625 ng/embryo (E), 0.125 ng/embryo (F), or 0.25 ng/embryo (G); and miR-145 mimic with three doses of *grnA* mRNA 0.1 ng/embryo (H), 0.2 ng/embryo (I), or 0.4 ng/embryo (J). (Scale bars, 100 μm; thirty embryos per experimental group were used and three independent replicates were performed).
(TIFF)

S5 Fig The regulatory mechanism between miR-145 and GrnA in hepatic outgrowth. (TIFF)

## Abbreviations

MiRNAs: microRNAs
GrnA: zebrafish progranulin A
PGRN: human progranulin
MET: hepatocyte growth factor receptor
WT: wild type
Tg: Transgenic line
EGFP: enhanced green fluorescent protein
ZFL cells: zebrafish normal liver cells
RT-PCR: reverse transcription polymerase chain reaction
PCNA: proliferating cell nuclear antigen
