# Supplementary figures and images for: MiR-145 mediates zebrafish hepatic outgrowth through progranulin A signaling
Source: PLoS One. 2017 May 22;12(5):e0177887. doi: 10.1371/journal.pone.0177887 (PMC5439702; doi:10.1371/journal.pone.0177887)

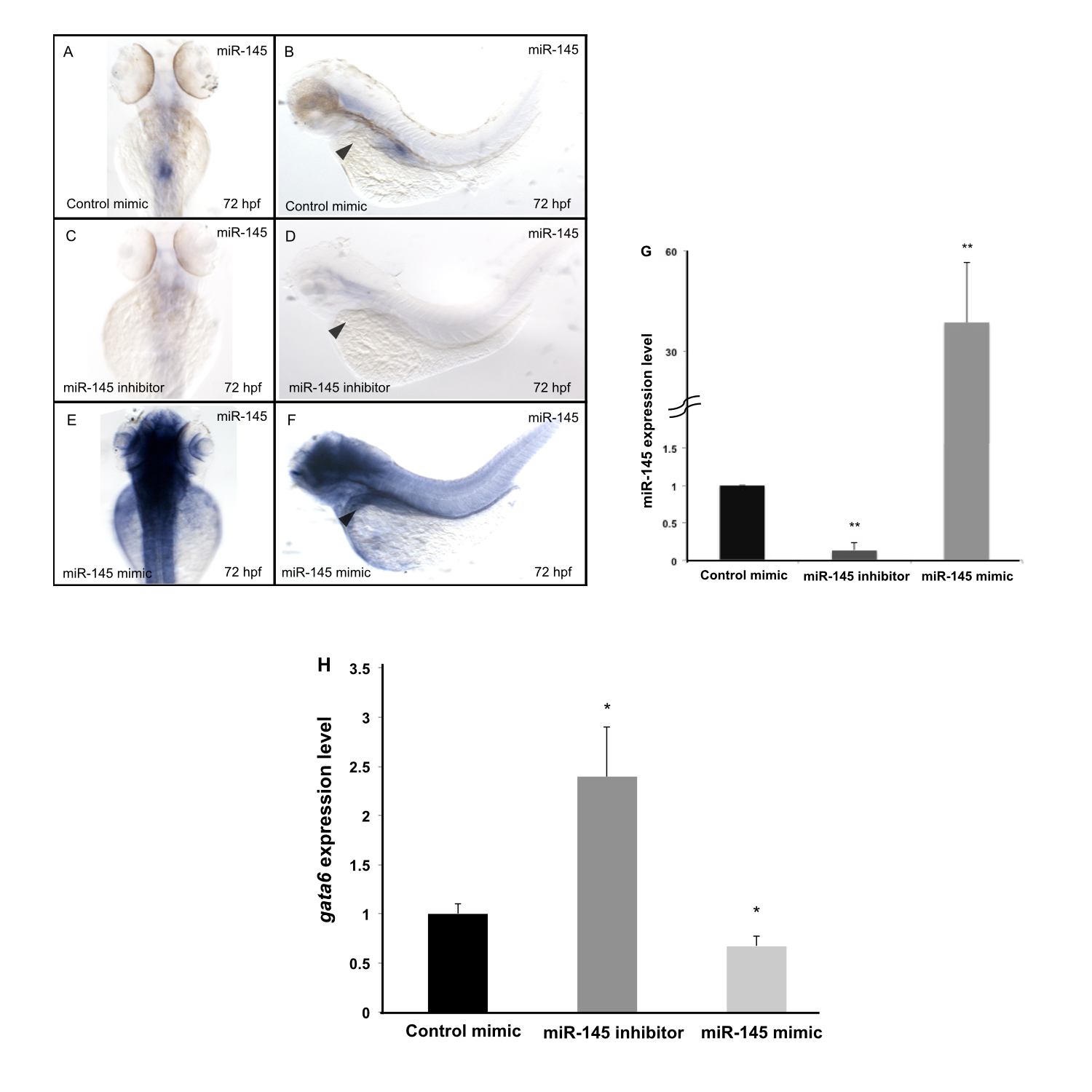

Supplement: S1 Fig — The miR-145 expression pattern at 72 hpf was examined using WISH and qPCR in control-, miR-145 inhibitor- and miR-145 mimic-injected fish. At 72 hpf, WISH indicates normal miR-145 expression in control-injected fish (A and B), reduced miR-145 expression in miR-145 inhibitor-injected fish (C and D) and overexpression of miR-145 in miR-145 mimic-injected fish (E and F). (A, C and E are dorsal views; B, D and F are lateral views). The qPCR analysis reveals that the miR-145 mimic and inhibitor modulate miR-145 expression (G). In addition, the qPCR analysis reveals that gata6 expression is regulated by altered expression of miR-145 (H). The ef1a and U6 expression was measured as a loading control. (**, P < 0.01, t-test; the arrow indicated liver, thirty embryos per experimental group were used and three independent replicates were performed). (TIFF) [file pone.0177887.s001.tiff]

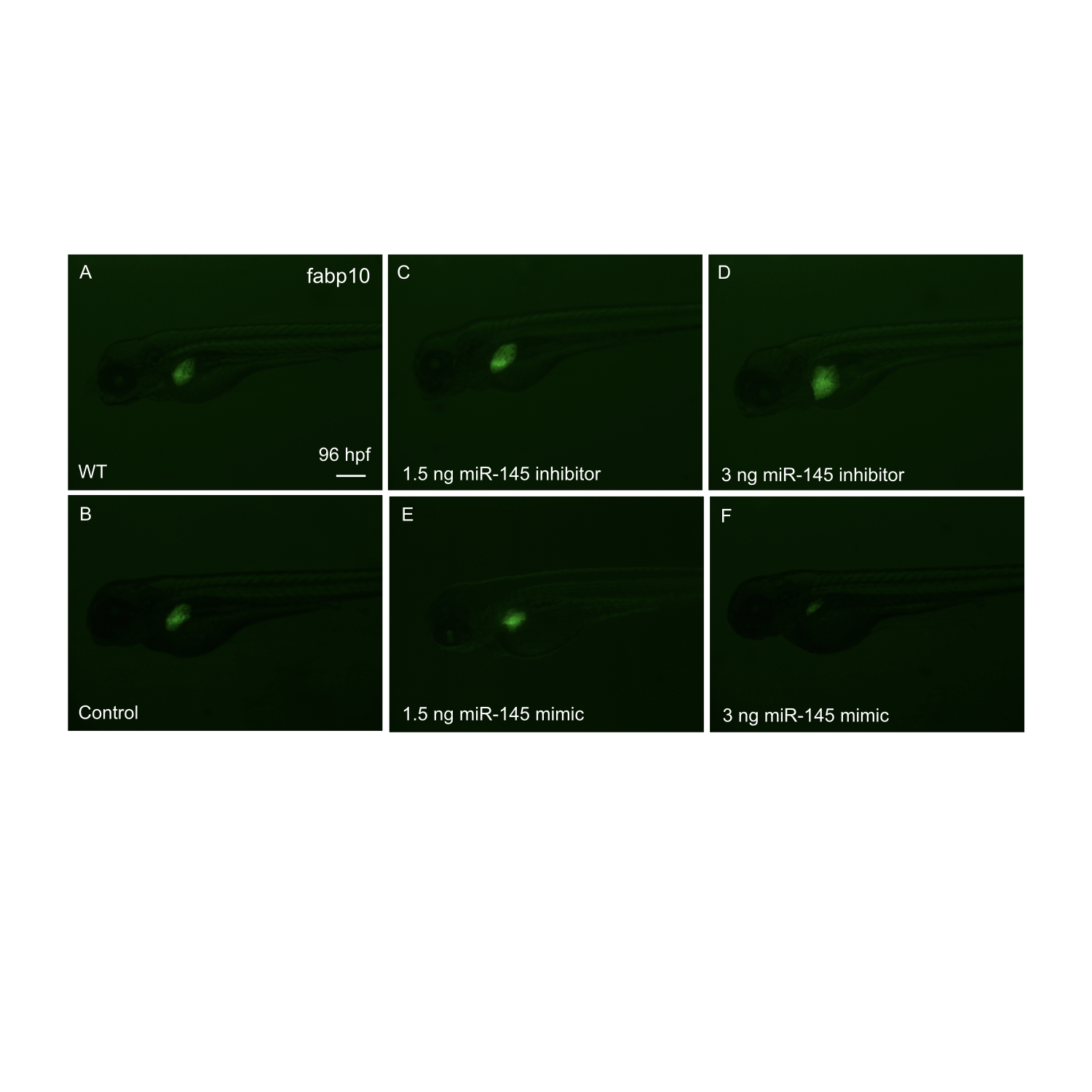

Supplement: S2 Fig — Liver morphology at 4 dpf after 1.5 ng and 3 ng control mimic, miR-145 inhibitor, miR-145 mimic injection in Tg(fabp10:EGFP) embryos. (Scale bars, 200 μm; EGFP, enhanced green fluorescent protein; thirty embryos per experimental group were used and three independent replicates were performed). (TIFF) [file pone.0177887.s002.tiff]

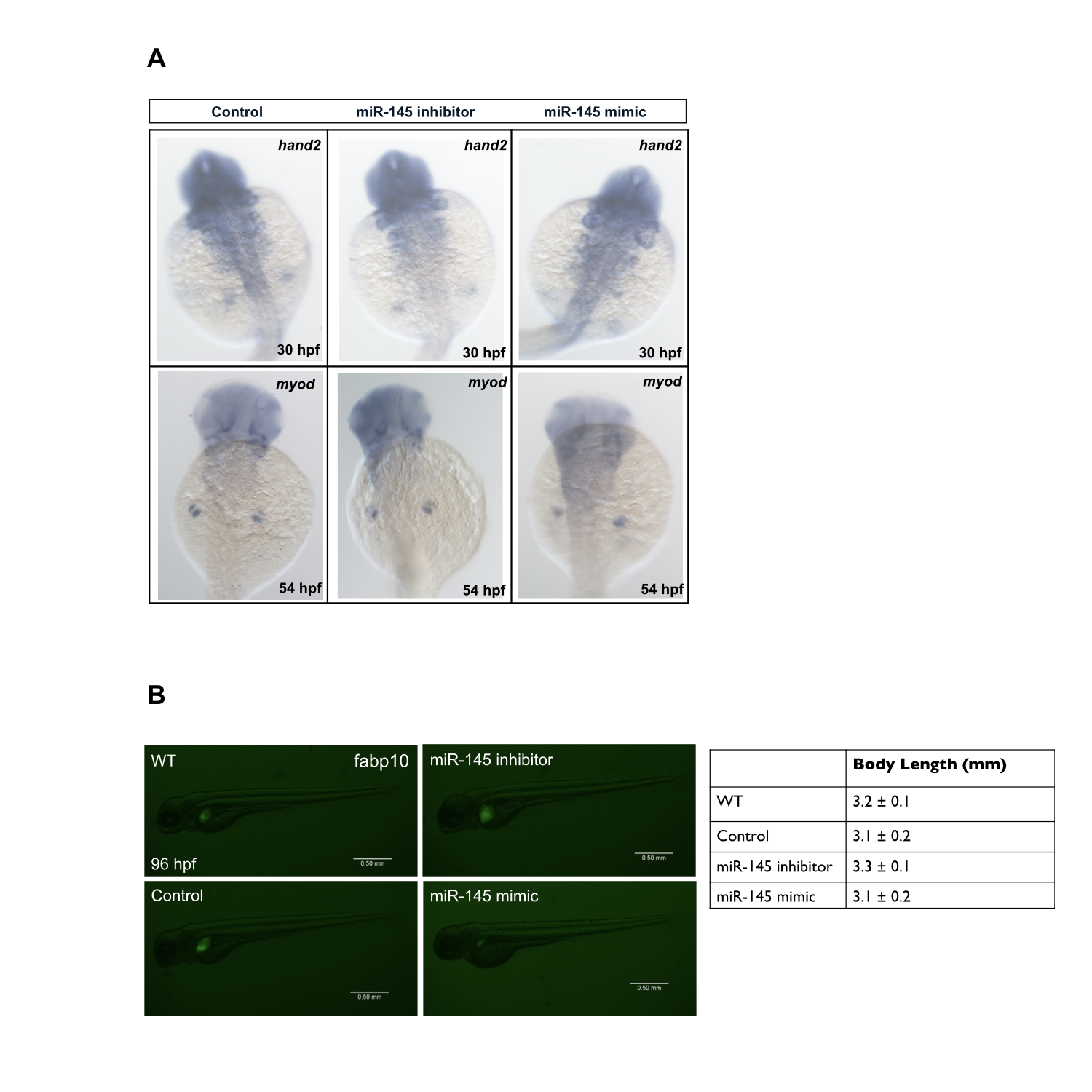

Supplement: S3 Fig — Two mesodermal genes, heart and neural crest derivatives expressed transcript 2 (hand2) at 30 hpf and myogenic differentiation 1(myod) at 54 hpf was examined using WISH. The result reveals mesoderm development was not significantly affected by manipulation of miR-145 expression (A). The body length were measured at 96 hpf after control mimic, miR-145 inhibitor, miR-145 mimic injection in Tg(fabp10:EGFP) embryos (B). (Scale bars, 0.50 mm; thirty embryos per experimental group were used and three independent replicates were performed). (TIFF) [file pone.0177887.s003.tiff]

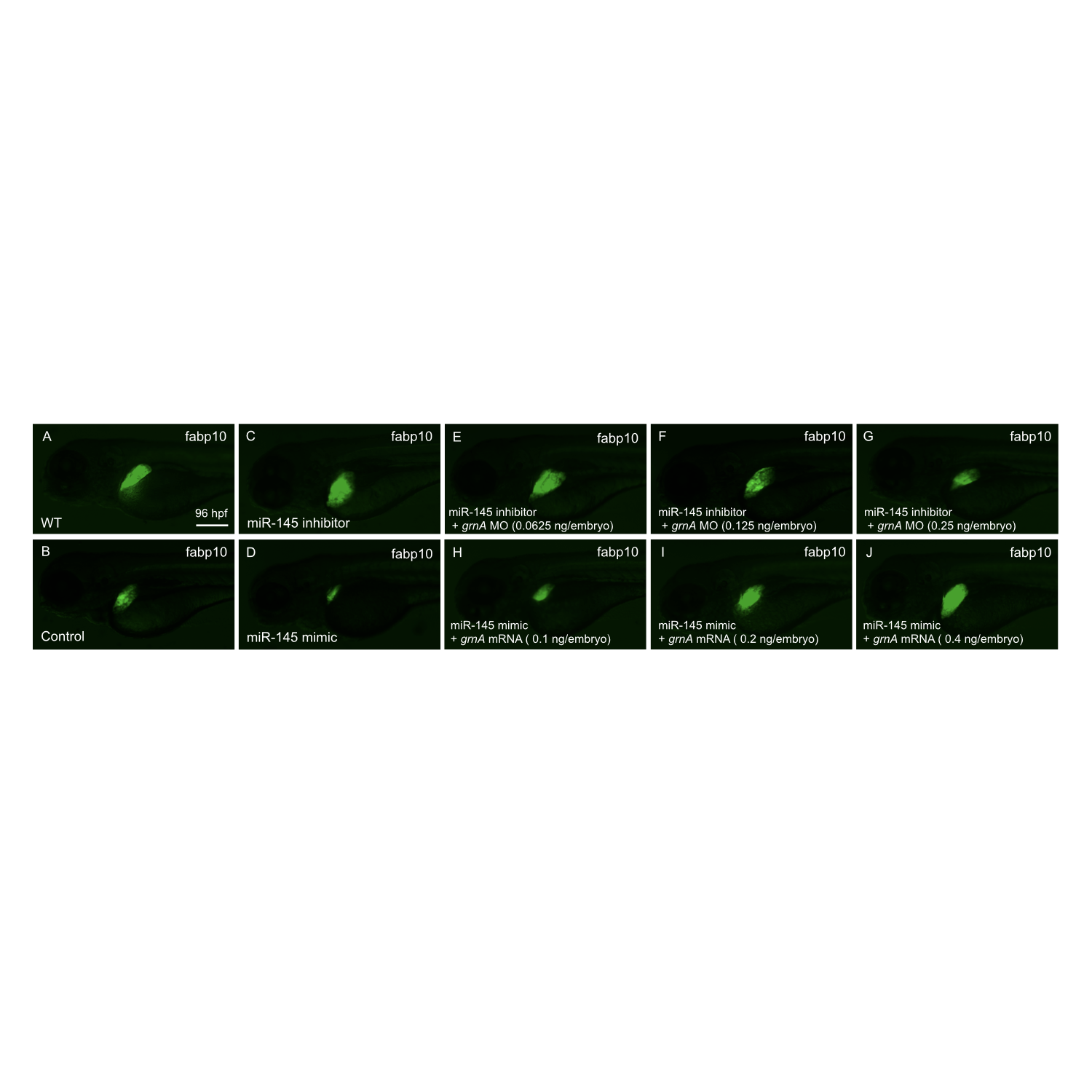

Supplement: S4 Fig — Liver morphology was determined by EGFP expression in Tg(fabp10:EGFP) embryos at 4 dpf (A) or in embryos injected with control mimic (B); miR-145 inhibitor (C); miR-145 mimic (D); miR-145 inhibitor with three doses of grnA MO 0.0625 ng/embryo (E), 0.125 ng/embryo (F), or 0.25 ng/embryo (G); and miR-145 mimic with three doses of grnA mRNA 0.1 ng/embryo (H), 0.2 ng/embryo (I), or 0.4 ng/embryo (J). (Scale bars, 100 μm; thirty embryos per experimental group were used and three independent replicates were performed). (TIFF) [file pone.0177887.s004.tiff]

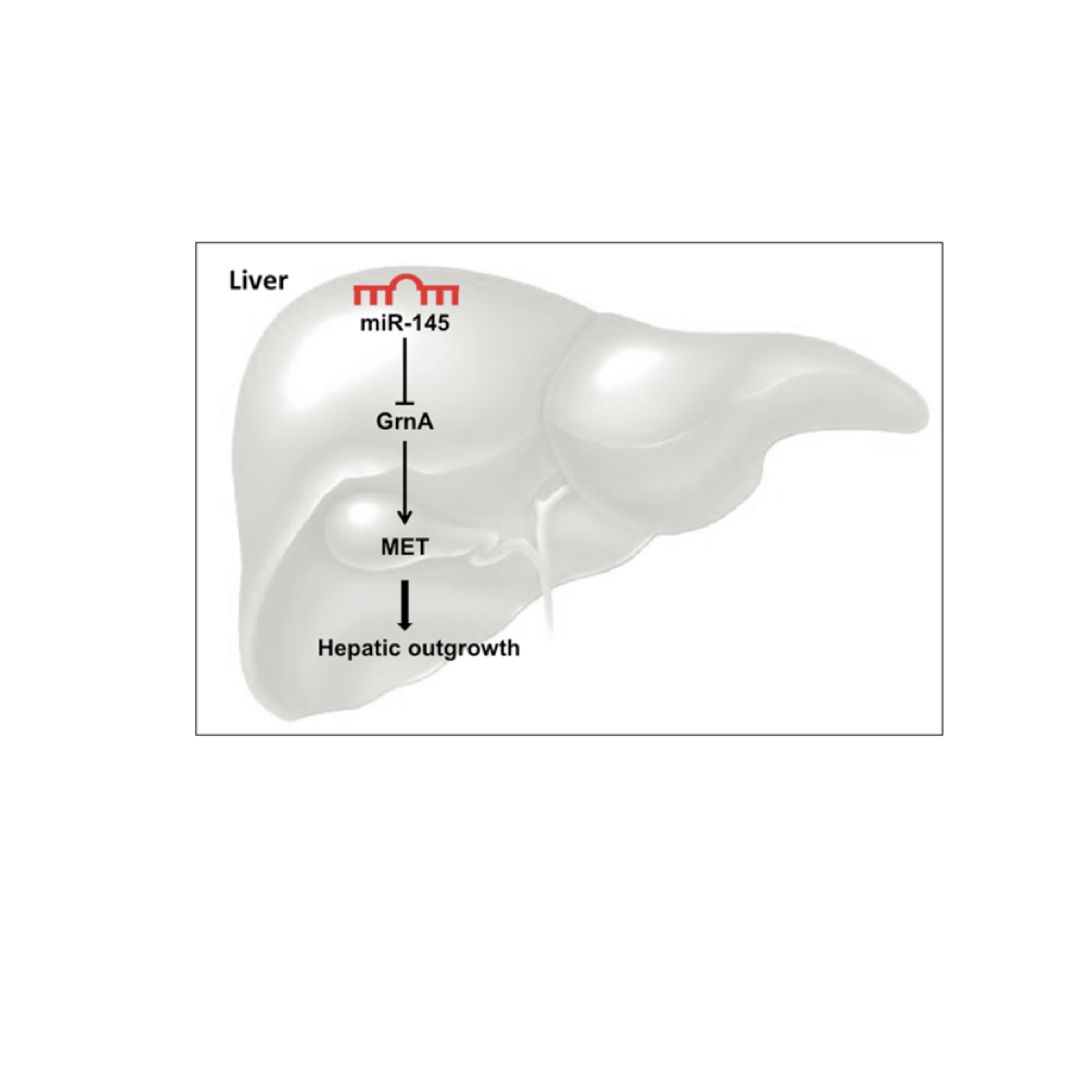

Supplement: S5 Fig — (TIFF) [file pone.0177887.s005.tiff]
